# 52 Co-development of the Physical Literacy Consensus Statement for England

**DOI:** 10.1093/eurpub/ckae114.048

**Published:** 2024-09-26

**Authors:** Lawrence Foweather, Mike Duncan, Inimfon Essiet, Liezel Hurter, Daniel Bingham, Jade Morris, Andy Daly-Smith, Will Roberts, Kiara Lewis, Hannah Goss, Wesley O’Brien, Cara Shearer, Lisa Barnett

**Affiliations:** Liverpool John Moores University, Liverpool, United Kingdom; Coventry University, Coventry, United Kingdom; Liverpool John Moores University, Liverpool, United Kingdom; Liverpool John Moores University, Liverpool, United Kingdom; University of Bradford, Bradford, United Kingdom; University of Bradford, Bradford, United Kingdom; University of Bradford, Bradford, United Kingdom; University of Waikato, Hamilton, New Zealand; Birmingham City University, Birmingham, United Kingdom; Dublin City University, Dublin, Ireland; University College Cork, Cork, Ireland; Liverpool John Moores University, Liverpool, United Kingdom; Deakin University, Melbourne, Australia

## Abstract

**Purpose:**

Physical literacy has gained considerable traction across physical activity, sport, health, and education sectors, leading to an abundance of definitions and interpretations worldwide. However, implementing and advocating for physical literacy becomes challenging when the concept holds different meanings for different individuals and organisations. ‘Uniting’ perspectives on the concept could catalyse efforts to adopt, support and promote physical literacy in practice. This study aimed to develop a physical literacy consensus statement for England that was accessible for those working in research, policy, and practice.

**Methods:**

Phase one included a review of the evidence, a first national stakeholder consultation, and focus groups with children and young people. Phase two included a modified-Delphi methodology and co-development of the draft statement with an expert panel of sixty researchers and stakeholders from around fifty organisations. Phase three included a second national consultation on the draft statement. Phase four involved further co-development and an online survey with the expert panel to revise the statement.

**Results:**

Consensus was established on a definition: Physical literacy is our relationship with movement and physical activity throughout life. The statement also included five key messages encompassing (1) Understanding physical literacy, (2) Why physical literacy matters, (3) Supporting physical literacy, (4) Our experiences affect our physical literacy, and (5) Physical literacy is personal.

**Conclusion:**

The consensus statement is for sector professionals and practitioners and provides a shared understanding of what physical literacy, why it is important and how it can be supported. By understanding, supporting, and adapting to the diverse needs and preferences of everyone, practitioners can play a pivotal role in enhancing physical literacy - an intrinsic driver for engagement in movement and physical activity behaviours. Future research should evaluate the implementation and the impact of the consensus statement.

**Funding:**

The National Lottery and Sport England

